# Manufacturing of Ti-Nb-Cr-V-Ni-Al Refractory High-Entropy Alloys Using Direct Energy Deposition

**DOI:** 10.3390/ma15196570

**Published:** 2022-09-22

**Authors:** Ho-In Jeong, Choon-Man Lee, Dong-Hyeon Kim

**Affiliations:** 1 Mechanical Design and Manufacturing, School of Mechatronics Engineering, Changwon National University, Changwon 51140, Korea; 2 Department of Mechanical Engineering, Changwon National University, Changwon 51140, Korea; 3 Mechatronics Research Center, Changwon National University, Changwon 51140, Korea

**Keywords:** additive manufacturing, direct energy deposition, high-entropy alloys, refractory material, phase analysis

## Abstract

High-entropy alloys (HEAs) are composed of 5–35 at% of five or more elements, have high configurational entropy, do not form intermetallic compounds, and have a single-phase face-centered cubic structure or body-centered cubic structure. In particular, refractory HEAs (RHEAs), based on refractory materials with excellent mechanical properties at high temperatures, have high strength and hardness at room temperature and excellent mechanical properties at low and high temperatures. In this study, the Ti-Nb-Cr-V-Ni-Al RHEAs were deposited using direct energy deposition (DED). In the microstructure of Ti-Nb-Cr-V-Ni-Al, the sigma, BCC A2, and Ti2Ni phases appeared to be different from the BCC A2, BCC B2, and Laves phases predicted in the phase diagram. This microstructure was similar to that of the casted Ti-Nb-Cr-V-Ni-Al and had a constructed fine grain size. It was found that the growth of these microstructures was due to the DED process, which has a fast solidification rate. The fine grain size caused high hardness, and the microhardness of the Ti-Nb-Cr-V-Ni-Al was measured to be about 900 HV. In addition, in order to analyze the thermal properties of Ti-Nb-Cr-V-Ni-Al composed of the refractory material, the heat-affected zone (HAZ) was analyzed through a preheating test. The HAZ was decreased, owing to the high thermal diffusivity of Ti-Nb-Cr-V-Ni-Al.

## 1. Introduction

High-entropy alloys (HEAs) are new materials that can overcome the limitations of the existing physical properties, and many studies are being conducted on them intensively [1]. General alloys are composed of the main element and a small number of substituted elements. In these alloys, as the number of constituent elements increases, intermetallic compounds are formed, and the mechanical properties are weakened, owing to their brittleness. HEAs are composed of 5–35 at% of five or more elements with similar atomic radii [2]. In this case, they exhibit high configurational entropy, severe lattice distortion, and a slow diffusion rate. In general, HEAs are defined as alloys with configurational entropies higher than 1.5R (where R is a gas constant) [3]. The configurational entropy equation is shown in Equation (1):

(1) $$\Delta S_{mix}=-R\sum_{i}^{N}x_{i}\ln x_{i},$$

where $\Delta s_{mix}\;$ is the configurational entropy, N is the number of elements, and $x_{i}$ is the entropy of each element.

Owing to their high configurational entropy, HEAs do not form intermetallic compounds and have a single-phase face-centered cubic (FCC) or body-centered cubic (BCC) structure [4]. Unlike the solid solution in a general alloy system, it forms a concentrated solid solution. Owing to their unique composition and microstructure, HEAs have not only high strength and hardness at room temperature but also excellent mechanical properties at low and high temperatures [5]. In particular, refractory high-entropy alloys (RHEAs) are the HEAs based on refractory materials such as Ti [6], V [7], Cr [8], Zr [9], Hf [10], Nb [11], Mo [12], Ta [13], W [14], and Mn [15], which have excellent mechanical properties at high temperatures. RHEAs have a crystal structure such as FCC, FCC+BCC, and BCC phases, a microstructure including a precipitated phase, and intermetallic compounds such as the BCC B2 phase and Laves phase [16]. Most research using HEAs was conducted during evaluation of the basic properties by manufacturing ingots using arc melting and casting [17]. The conventional casting method of manufacturing HEAs has the problem of difficulty in forming a single-phase solid solution due to a low cooling rate. As the size of the manufactured HEA increases, it is difficult to secure the uniformity of the microstructure because the internal cooling rate and the external cooling rate are different. The non-uniformed microstructure causes degradation and non-uniformity of the mechanical properties of the HEAs [18,19]. In addition, since the HEAs are challenging to process due to their high mechanical properties, research is needed to reduce the material’s volume and improve the substrate’s performance by coating the base material with HEAs rather than manufacturing a large volume. Therefore, research on the manufacture of HEAs using additive manufacturing (AM) that can be manufactured and coated in a relatively short time is needed.

The AM process’s research and development has been actively conducted because it has the advantages of easy design and manufacturing of complex or special shapes, and it is used in various fields such as aerospace, automobiles, medicine, and machinery. AM technology can be classified by processes into two types: the powder bed fusion (PBF) process and directed energy deposition (DED) process [20]. PBF is an AM process in which thermal energy (a laser or electron beam) selectively fuses regions of a powder bed layer by layer [21]. PBF can create arbitrarily complex geometries by selectively melting powder-form raw materials using a bottom-up manner with the assistance of a thermal energy source [22], and it shows overwhelming superiority in the manufacture of objects with geometrical complexity and high resolutions [23]. DED is an AM process in which metal wire or powder is combined with a thermal energy source to directly deposit material onto a build tray or an existing part. The DED method is similar to welding and can be used to deposit material on existing products, thus being available for maintenance work. In addition, in the DED process, since AM of multi-material objects is possible, various alloys and composites can be manufactured using various metal materials. Therefore, AM, which is completed in a relatively short time and manufactured using various metal materials, is suitable for manufacturing HEAs. DED has mostly been used in research on manufacturing HEAs using additive manufacturing [24]. Because DED has a fast solidification rate, it is advantageous for refining grains and can prevent the segregation of specific components [25]. In addition, because the heat source has a high energy density, the heat-affected zone (HAZ) is narrow, and the dilution rate of the base material is low. These features mainly reduce cracks, deformations, and metallurgical changes in the base metal, resulting in fewer defects and better production quality than other methods [26].

**Figure 1:**
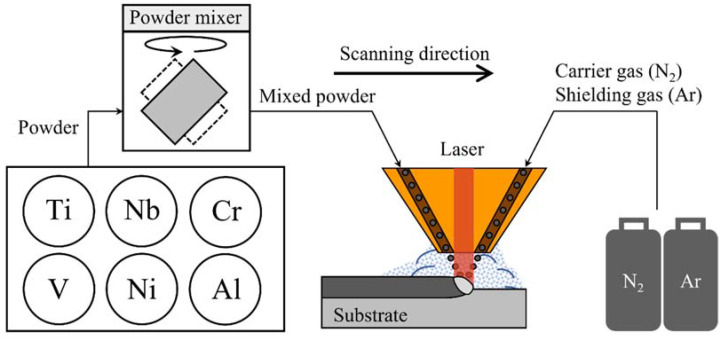
Schematic diagram for DED of Ti-Nb-Cr-V-Ni-Al.

In this study, a Ti-Nb-Cr-V-Ni-Al RHEA was deposited using DED. Figure 1 shows the schematic diagram for the DED of Ti-Nb-Cr-V-Ni-Al. Prior to deposition, the phase diagram and phase fraction of Ti-Nb-Cr-V-Ni-Al were analyzed. For the DED of Ti-Nb-Cr-V-Ni-Al, powders of each element were mixed to prepare a mixed Ti-Nb-Cr-V-Ni-Al powder and deposited on Ti-6Al-4V and Inconel 718 substrates. The scanning electron microscopy (SEM), electron backscatter diffraction (EBSD) X-ray diffraction (XRD), and energy dispersive spectrometer (EDS) analyses were conducted to verify the microstructure of the deposited Ti-Nb-Cr-V-Ni-Al. In addition, the hardness of the deposited Ti-Nb-Cr-V-Ni-Al was measured, and a preheating test was performed to analyze the HAZ depending on the deposited Ti-Nb-Cr-V-Ni-Al.

## 2. Phase Analysis

**Figure 2:**
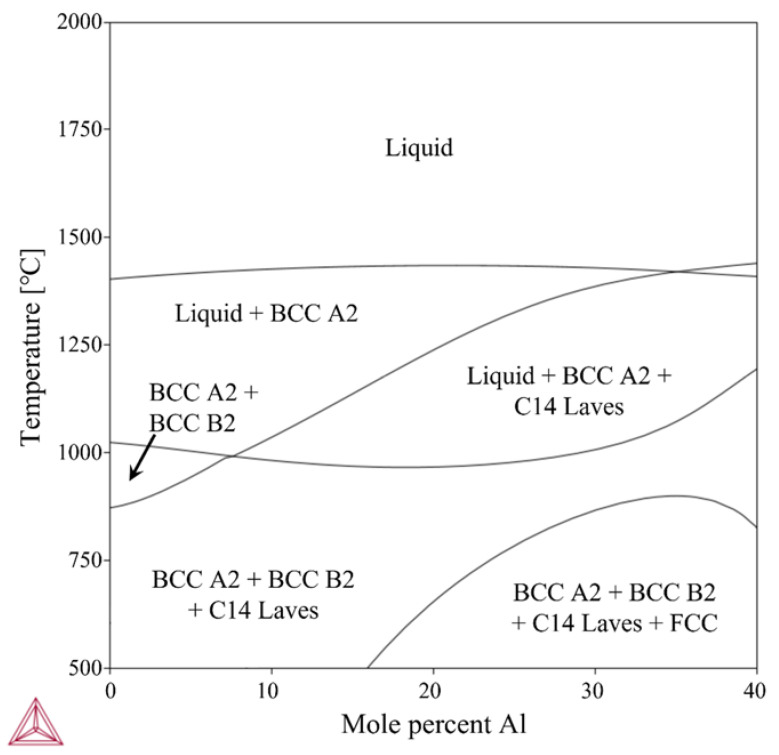
The phase diagram of Ti-Nb-Cr-V-Ni-Al.

**Figure 3:**
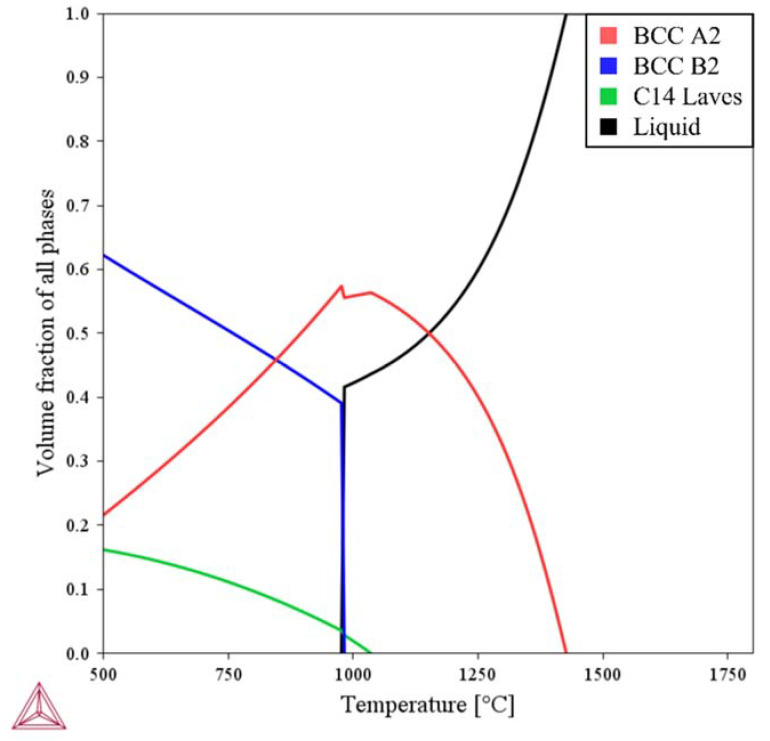
The phase fractions of Ti-Nb-Cr-V-Ni-Al.

Table 1 lists chemical composition of Ti-Nb-Cr-V-Ni-Al. The Ti-Nb-Cr-V-Ni-Al was completely melted at above 1425 °C, and the Ti-rich BCC A2 phase grew as the temperature decreased. The BCC A2 phase increased up to a complete solidification temperature of 950 °C and decreased rapidly with the formation of the Ti–Nb-rich BCC B2 precipitated phase at 950 °C. The Cr–Nb-rich C14 Laves phase as a precipitate phase appeared at 1030 °C and gradually grew with decreasing temperature. The predicted phase fractions were BCC A2 (21.6%), BCC B2 (62.2%), and BCC C14 Laves (16.2%). Figure 2 shows the phase diagram of Ti-Nb-Cr-V-Ni-Al, and Figure 3 shows the phase fractions of Ti-Nb-Cr-V-Ni-Al.

The growth of the BCC phase was found to be due to the beta (β) phase of titanium. Titanium was encountered in two crystallographic forms. At room temperature, unalloyed titanium has a hexagonal close-packed (HCP) structure referred to as the alpha (α) phase. At 883 °C, titanium undergoes an allotropic transformation from HCP to the BCC structure known as the β phase. The α phase generally has creep resistance superior to the β phase and is preferred for high-temperature applications. The absence of a ductile-to-brittle transition, a feature of the β phase, makes the α phase suitable for cryogenic applications. The β phase has better strength and toughness than the α phase. They also exhibit high-stress corrosion resistance, can be heat-treated to high strengths, and have excellent fatigue properties [27]. In addition, most elements constituting Ti-Nb-Cr-V-Ni-Al, such as Nb, Cr, V, and Cr as the transition elements, decrease the temperature of the α-to-β phase transition and thus promote the development of the β phase [28].

## 3. Experimental Procedure

### 3.1. Experimental Set-Up and Materials

**Figure 4:**
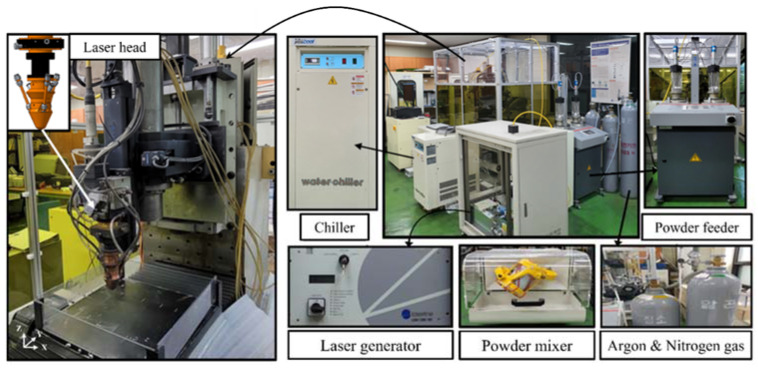
The experimental set-up for DED.

To perform DED of Ti-Nb-Cr-V-Ni-Al, a hybrid machine tool for additive and subtractive manufacturing was used with a high-power diode laser additive head (Laytools, AK390) [29]. The hybrid machine tool had a travel distance of 500 × 500 × 300 mm (X, Y, Z). A laser (Laserline, LDM 1000) with a maximum output of 1 kW, wavelength of 980 nm, focal length of 198 mm, and spot size of 3 mm was used for the laser head [30]. The powders were mixed using a mixer for 4 h and transferred to a laser head using a rotating twin disk-type powder feeder system (Oerlikon Metco, Twin 150). Nitrogen was used as the carrier gas for powder feeding, and argon gas was used as the shield gas [31]. A 780-W cooling system (Yescool, YRC-1A) was used to cool the hybrid machine tool. Figure 4 shows the experimental set-up.

The powder used for the DED of Ti-Nb-Cr-V-Ni-Al was prepared by mixing Ti, Nb, Cr, V, Ni, and Al powders manufactured by MK Corporation in the particle size range of 45–150 μm. The powder was mixed at the same ratio as in the phase analysis. The substrates used were 15 mm wide, 15 mm thick, and 60 mm long for Ti-6Al-4V and Inconel 718 to prevent thermal deformation and thermal shock during DED. Table 2 lists the chemical compositions of the Ti-6Al-4V and Inconel 718 substrates [32,33].

### 3.2. DED Method and Conditions

**Figure 5:**
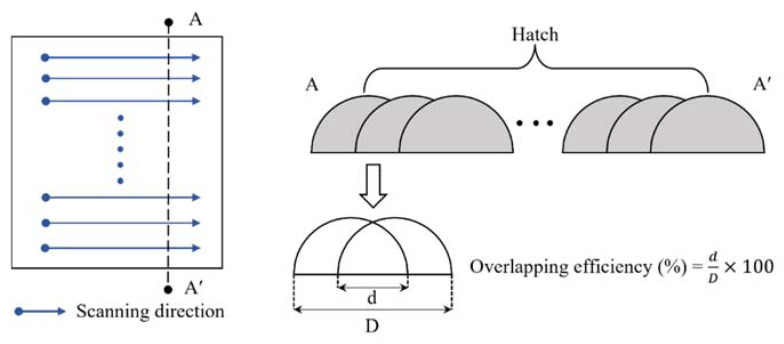
The schematic diagram of the overlapping efficiency.

**Figure 6:**
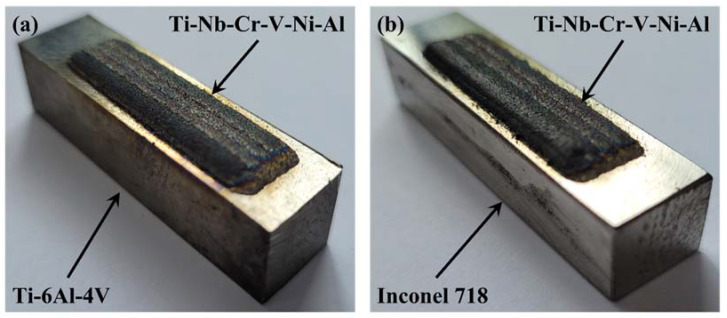
The results for Ti-Nb-Cr-V-Ni-Al: (**a**) Ti-Nb-Cr-V-Ni-Al deposited on Ti-6Al-4V and (**b**) Ti-Nb-Cr-V-Ni-Al deposited on Inconel 718.

Ti-Nb-Cr-V-Ni-Al was deposited on Ti-6Al-4V and Inconel 718 substrates in a rectangular shape of 10 mm transversely (X direction) and 40 mm longitudinally (Y direction). The Ti-Nb-Cr-V-Ni-Al was deposited in two layers to a thickness of 1 mm. Each layer was deposited with five hatches, and the overlapping efficiency of the hatch was 40%. If the efficiency was lower than 30%, internal pores may have been generated, and the mechanical properties may have been reduced [20,34]. If the overlapping efficiency was too high, deformation and internal stress would occur, owing to the reduced productivity and thermal overlap. Figure 5 shows the schematic diagram of the overlapping efficiency. In the deposition of Ti-Nb-Cr-V-Ni-Al, the laser power, scan speed, powder feed rate, and shield gas flow rate were used as deposition parameters. The experiment was conducted at room temperature and in a habitual state, and the DED equipment was sealed to block the inflow of air and prevent oxidation. Table 3 lists the deposition conditions, and Figure 6 shows the DED results of Ti-Nb-Cr-V-Ni-Al.

## 4. Results

### 4.1. Microstructure

**Figure 7:**
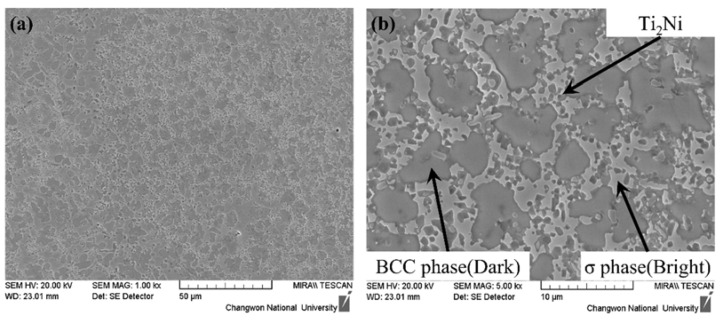
The SEM image of the Ti-Nb-Cr-V-Ni-Al: (**a**) ×1000 magnification and (**b**) ×5000 magnification.

**Figure 8:**
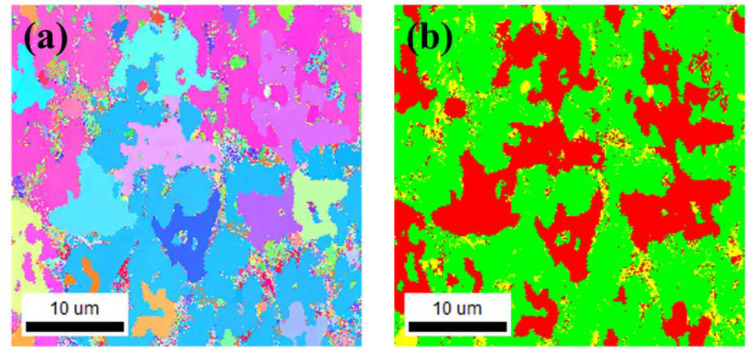
The EBSD results of the Ti-Nb-Cr-V-Ni-Al: (**a**) IPF map and (**b**) phase map (red = BCC A2, green = σ, and yellow = Ti_2_Ni).

To investigate the microstructure of the deposited Ti-Nb-Cr-V-Ni-Al, SEM images and EBSD analyses were performed. To fabricate the SEM and EBSD specimens, 5 × 5 × 5 mm Ti-Nb-Cr-V-Ni-Al was deposited on a Ti-6Al-4V substrate. To prevent the inflow of components of the substrate through the melting pool, a 3 × 3 × 3 mm specimen was fabricated by cutting the deposited Ti-Nb-Cr-V-Ni-Al with a wire, and the deposited specimens were subjected to mechanical polishing for analysis. From the phase analysis, it was predicted that the BCC B2 phase was 62.2%, the BCC A2 phase was 21.6%, and the C14 Laves phase was 16.2% (Figure 3). However, the sigma(σ) and Ti_2_Ni phases appeared in the deposited Ti-Nb-Cr-V-Ni-Al [35,36]. Microstructure analysis revealed that the σ phase appeared in a form surrounding the BCC A2 phase, and the Ti_2_Ni phase appeared in a fine form inside the σ and BCC A2 phases [6,37]. The microstructure of Ti-Nb-Cr-V-Ni-Al showed that the BCC A2 phase, which grew first at high temperatures, grew first, and then the σ phase grew rapidly before the Ti_2_Ni precipitated phase grew [38,39]. Figure 7 shows the SEM image of Ti-Nb-Cr-V-Ni-Al. EBSD analysis revealed that the fraction of the BCC A2 phase was 28.2%, that of the σ phase was 64.2%, and that of the Ti_2_Ni phase was 7.6%. Figure 8a shows the IPF map of Ti-Nb-Cr-V-Ni-Al, and Figure 8b shows the phase map of Ti-Nb-Cr-V-Ni-Al. The microstructure of the deposited Ti-Nb-Cr-V-Ni-Al contained the σ phase, which was not predicted by the phase analysis. The reason for the appearance of unexpected phases from the phase analysis is that the accuracy of the phase analysis is highly dependent on the thermodynamic database used, and a complete thermodynamic analysis of each binary and ternary phase diagram belonging to the multicomponent system was not performed for accurate calculations [40,41,42]. However, the microstructure was similar to that of Ti-Nb-Cr-V-Ni-Al that was heat-treated after casting [6]. It was found that the growth of this microstructure was because of the high energy density of the laser used during DED, which rapidly increased the temperature to a high temperature and then showed the heat treatment effect because of the rapid cooling. Rapid temperature changes also affected the grain size. The grain size of the Ti-Nb-Cr-V-Ni-Al was 1–35 μm, which was finer than the 45–150 μm powder size used.

### 4.2. XRD and EDS Analyses

**Figure 9:**
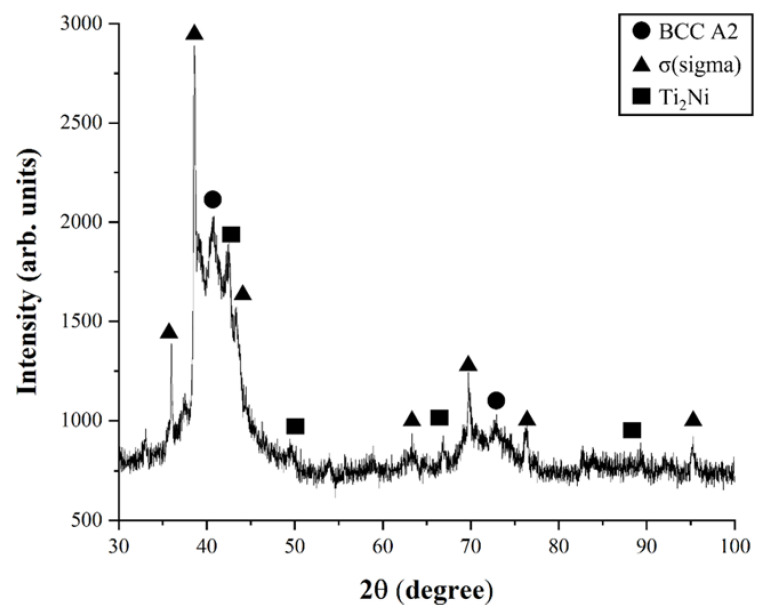
The results of XRD analysis of Ti-Nb-Cr-V-Ni-Al.

The XRD and EDS analyses were performed to verify the phase and components of Ti-Nb-Cr-V-Ni-Al. Figure 9 shows the results of the XRD analysis. In the results of the XRD analysis, the σ phase peak was higher than those of the BCC A2 and Ti_2_Ni phases. As a result of the XRD analysis, the peaks for the three phases occurred as shown in the phase map, where the intensity of the σ phase peak was the highest and the intensity of the Ti_2_Ni phase peak was the lowest. Additionally, a lot of noise was generated as a result of the XRD analysis. It was found that the noise was caused by not melting the powder inside the deposited Ti-Nb-Cr-V-Ni-Al. In order to reduce this noise, it was confirmed that the deposit should be performed at a higher temperature so that the powder could be completely melted.

The EDS analysis was analyzed through SEM imaging (Figure 7b). For component analysis of Ti-Nb-Cr-V-Ni-Al and each phase, component analysis of the entire area and each phase point was conducted. Table 4 shows the chemical compositions of the structure constituents for Ti-Nb-Cr-V-Ni-Al. As a result of EDS analysis, the BCC A2 phase showed a Ti-Nb-rich composition, as in the phase analysis results. The σ phase, which did not appear as a result of the previous phase analysis, showed a Ti-Ni-rich composition, as in the BCC B2 phase. The Ti_2_Ni phase showed the highest Ti composition and showed the Ti_2_Ni ratio composition. The composition of Ti-Nb-Cr-V-Ni-Al was similar to the design composition, but the composition of Nb was measured to be slightly lower.

### 4.3. Microhardness

**Figure 10:**
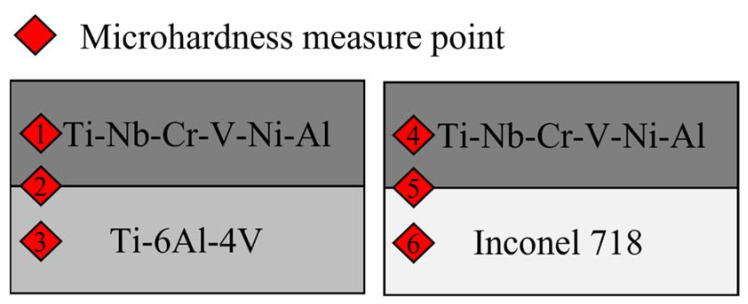
The measure point for the microhardness of Ti-Nb-Cr-V-Ni-Al deposited on the substrates.

**Figure 11:**
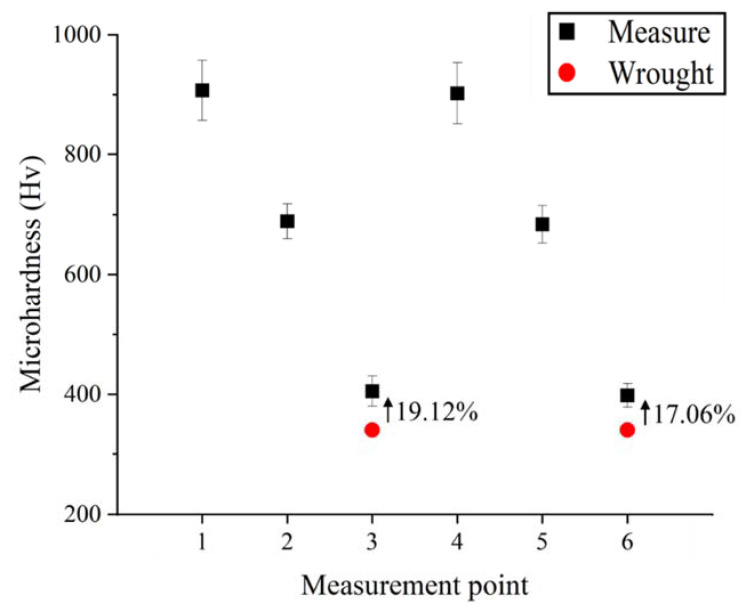
The microhardness distributions of Ti-Nb-Cr-V-Ni-Al deposited on the substrates.

The microhardness measurements were performed for Ti-Nb-Cr-V-Ni-Al deposited by DED. To measure the microhardness as shown in Figure 10, two Ti-Nb-Cr-V-Ni-Al specimens deposited on Ti-6Al-4V and Inconel 718 were divided into three sections: a deposition area, a substrate area, and a bonding layer for a total of six Vickers hardness (Hv) areas measured. The microhardness measurement was performed nine times for each area to consider the non-uniformity of the hardness according to the deposition height, and the average value was used as the result. The microhardness of the Ti-Nb-Cr-V-Ni-Al deposited area was measured to be approximately 900 HV. The microhardness of Ti-6Al-4V and Inconel 718 used by the substrates increased by 17–19% compared with that of the wrought material (340 Hv), and the microhardness of the bonding layer was measured to be an intermediate value between the microhardness of the deposition area and the substrate area [43,44]. Figure 11 shows the microhardness distribution. The high microhardness of Ti-Nb-Cr-V-Ni-Al was related to the high volume fraction of the σ phase. The σ phase is known to exhibit brittleness and high hardness. Therefore, the high volume fraction of the σ phase for Ti-Nb-Cr-V-Ni-Al increased the hardness and decreased the ductility. The cause of the increased microhardness of the substrate was found to be heat treatment, owing to the rapid temperature change occurring in the DED.

### 4.4. Heat-Affected Zone

**Figure 12:**
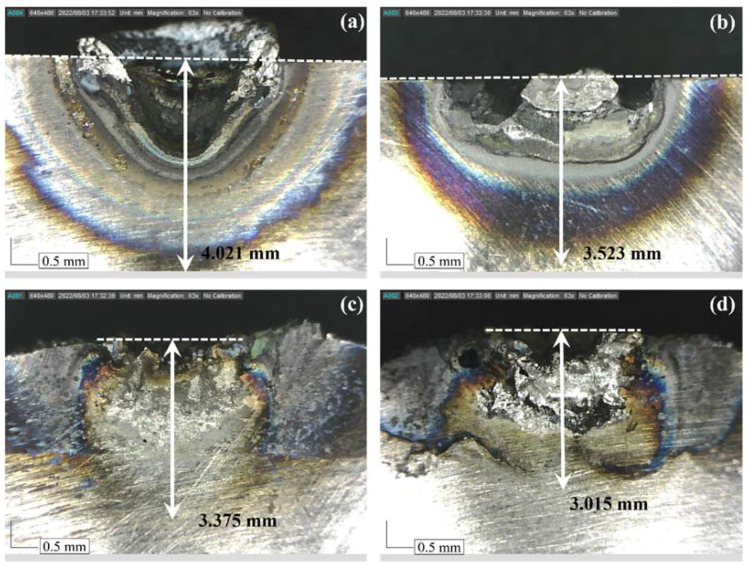
The measurement results of the HAZ: (**a**) Ti-6Al-4V, (**b**) Inconel 718, (**c**) Ti-Nb-Cr-V-Ni-Al deposited on Ti-6Al-4V, and (**d**) Ti-Nb-Cr-V-Ni-Al deposited on Inconel 718.

To analyze the thermal properties and thermal shielding effect of Ti-Nb-Cr-V-Ni-Al composed of the refractory material, the HAZ was analyzed through a preheating test. For HAZ measurement, the surface of the specimen was preheated with a laser with a 500-W output and 15 L/min shield gas (Ar) for 3 s [45]. Preheating tests were performed on four specimens of conventional Ti-6Al-4V and Inconel 718 as well as Ti-6Al-4V and Inconel 718 deposited with Ti-Nb-Cr-V-Ni-Al. Figure 12 shows the measurement results for the HAZ depth for each specimen. The HAZ depth of the conventional Ti-6Al-4V was 4.021 mm (Figure 12a), and the HAZ depth of the Ti-Nb-Cr-V-Ni-Al-deposited Ti-6Al-4V was 3.375 mm (Figure 12c). The HAZ depth of the conventional Inconel 718 was 3.523 mm (Figure 12b), and the HAZ depth of the Inconel 718 deposited with Ti-Nb-Cr-V-Ni-Al was 3.015 mm (Figure 12d). The HAZ size was determined by the thermal diffusivity of each material. The thermal diffusivity of the Inconel 718 was higher than that of the Ti-6Al-4V, and the HAZ was smaller in the Inconel 718 [46,47]. The HAZ depths of the Ti-6Al-4V and Inconel 718 were reduced by 16.07% and 14.42%, respectively, according to the Ti-Nb-Cr-V-Ni-Al deposition. As a result of the HAZ measurement, it was found that the Ti-Nb-Cr-V-Ni-Al had a higher thermal diffusivity than the Inconel 718 and that the thermal shielding effect could be obtained through the deposition of Ti-Nb-Cr-V-Ni-Al.

## 5. Conclusions

In this study, a Ti-Nb-Cr-V-Ni-Al RHEA was deposited using DED. The phase diagram and phase fraction of Ti-Nb-Cr-V-Ni-Al were analyzed for deposition on Ti-Nb-Cr-V-Ni-Al. For the deposited Ti-Nb-Cr-V-Ni-Al, powders of each element were mixed to prepare a mixed Ti-Nb-Cr-V-Ni-Al powder and deposited on Ti-6Al-4V and Inconel 718 substrates. The microstructure and hardness of the deposited Ti-Nb-Cr-V-Ni-Al were measured. In addition, HAZ and thermal shielding analyses were performed on the deposited Ti-Nb-Cr-V-Ni-Al. The primary findings of this study are as follows:

- SEM images and EBSD analysis were used to investigate the microstructure of the deposited Ti-Nb-Cr-V-Ni-Al. Phase analysis predicted that the BCC B2 phase was 62.2%, the BCC A2 phase was 21.6%, and C14 Laves phase was 16.2%. However, the σ phase was 64.2%, the BCC A2 phase was 28.2%, and the Ti_2_Ni phase was 7.6% in the deposited Ti-Nb-Cr-V-Ni-Al.
- The microstructure of the deposited Ti-Nb-Cr-V-Ni-Al consisted of a phase not predicted by the phase analysis but showed a microstructure similar to that of Ti-Nb-Cr-V-Ni-Al annealed after casting. In addition, the deposited Ti-Nb-Cr-V-Ni-Al exhibited a fine grain size of 1–35 μm. The growth of this microstructure was attributed to the high energy density of the laser used in DED, which showed the heat treatment effect by rapid cooling after rapidly increasing the temperature to a high temperature.
- The microhardness of the deposited Ti-Nb-Cr-V-Ni-Al was investigated. The microhardness of the Ti-Nb-Cr-V-Ni-Al deposited area was measured to be approximately 900 HV. The microhardness of the Ti-6Al-4V and Inconel 718 used by the substrates increased by 17–19% compared with that of the wrought material, and the microhardness of the bonding layer was measured to be an intermediate value of the microhardness of the deposition area and the substrate. The cause of the increased microhardness of the substrate was analyzed as heat treatment, owing to the rapid temperature change occurring in the DED.
- A preheat test was performed for the HAZ measurement of the Ti-Nb-Cr-V-Ni-Al deposited by DED. The HAZ depths of the Ti-6Al-4V and Inconel 718 were reduced by 16.07% and 14.42%, respectively, according to the Ti-Nb-Cr-V-Ni-Al deposition. The HAZ decreased owing to the high thermal diffusivity of Ti-Nb-Cr-V-Ni-Al. In addition, the deposited Ti-Nb-Cr-V-Ni-Al exhibited a heat-shielding effect.

The RHEAs had excellent mechanical properties at high temperatures, so their usefulness is high. However, it is necessary to study the manufacturing method because the design of the material is difficult due to the correlation between the constituent materials. In particular, since additive manufacturing of RHEAs has many process variables and rapid temperature changes occur, it is necessary to analyze the effect of each process variable on phase formation. In this study, it was found that an unexpected phase occurred in the preparation of RHEAs using DED. In future research, we will analyze the effect of each process variable on phase formation and find suitable manufacturing conditions for RHEAs by controlling the process variables. This study on the manufacturing of RHEAs with a Ti-Nb-Cr-V-Ni-Al composition using DED can be used as a novel RHEA manufacturing method and can be used as basic data for future research of RHEA phase analysis according to additive manufacturing conditions.

## Figures and Tables

**Table 1:** The chemical composition of Ti-Nb-Cr-V-Ni-Al.

Element	Ti	Nb	Cr	V	Ni	Al
at %	34.6	20.4	10.0	10.0	15.0	10.0

**Table 2:** The chemical compositions of the substrates.

**Ti-6Al-4V**												
Element	Ti		Al			V		Fe			C	
wt %	Bal.		6.5			4.3		0.15			0.02	
**Inconel 718**												
Element	Ni	Cr		Fe	Nb		Mo		Ti	Al		Co
wt %	Bal.	18.1		17.9	4.8		2.9		0.9	0.5		0.3

**Table 3:** The DED conditions.

Laser Power (W)	Scanning Speed (mm/s)	Powder Feed Rate (g/min)	Shield Gas Flow Rate (L/min)
1000	8	13	15

**Table 4:** The chemical compositions of the structure constituents for Ti-Nb-Cr-V-Ni-Al.

Element (at %)	Ti	Nb	Cr	V	Ni	Al
Constituents						
BCC A2 phase	38.4	28.7	9.0	10.1	6.0	7.8
σ phase	32.3	15.1	13.9	9.0	21.1	8.6
Ti_2_Ni phase	46.2	8.2	7.0	8.8	23.8	6.0
Ti-Nb-Cr-V-Ni-Al	37.5	13.2	8.8	14.5	16.7	9.3
Nominal composition	34.6	20.4	10.0	10.0	15.0	10.0

## Data Availability

Data presented in this article are available upon request from the corresponding author.
